# Commentary on “Duox-driven ROS release by glia promotes regeneration in the adult Drosophila brain”

**DOI:** 10.1080/19336934.2026.2689814

**Published:** 2026-07-01

**Authors:** Eli C. Scott, Grace Boekhoff-Falk

**Affiliations:** Department of Cell and Regenerative Biology, University of Wisconsin-Madison, School of Medicine and Public Health, Madison, WI USA

**Keywords:** Development, neurogenesis, neural development, stem cells, signalling

The recent report by Alves et al. 2026, [1] exploits a model first described by Fernandez-Hernandez et al. in 2013 in which penetrating injury to the optic lobe of the adult Drosophila brain results in cell proliferation and the generation of new neurons. In the original study, the proliferating cells were identified as deadpan-expressing quiescent neural progenitors (NPs) and shown to give rise to new neurons [2]. The new paper presents a convincing argument that glial-derived reactive oxygen species (ROS) stimulate proliferation following injury to the adult optic lobes (Fig. 2P–T, 3E–I) but also leaves some questions unanswered as to what cells are proliferating and by what mechanisms proliferation occurs.

The authors first provide data that penetrating injury to the optic lobe of the adult Drosophila brain leads to the production of both glia-derived ROS and glial expression of genes whose products are involved in scavenging ROS (Fig. 1D–N) [1]. They also provide evidence that ROS promote c-Jun N-terminal kinase (JNK) activation (Fig. 4K–L) followed by cell proliferation in the Drosophila optic lobe (Fig. 2A–C, S–T). One of the more surprising findings is that although most ROS are produced by oxidative phosphorylation in mitochondria [3], it is ROS produced at the membrane by Dual oxidase (Duox) that is required for the brain’s proliferative response (Fig. 2P-T). Indeed, if *duox* activity is globally reduced via RNAi, neither ROS production nor cell proliferation is observed post-injury (Fig. 2F–H, P–S). Glial-specific knockdown of *duox* gives similar results (Fig. 2T). Consistent with the genetic data, feeding flies the ROS scavenger N-Acetylcysteine (NAC) also prevents cell proliferation post-injury (Fig. 3E-I).

To gain insights into the molecular mechanisms underlying the injury response, the authors investigated the roles of calcium and JNK signalling in post-injury proliferation. While both are upregulated by injury (Fig. 4B–G, H–J), the requirement for JNK signalling in stimulating the proliferative response also is reported (Fig. 1B–C, Figure 2P–T, Figure 4N–Q, S). Additionally, the authors report that JNK signalling is elevated after injury in both neurons and glia (Fig. 4 R). Based on the experiments presented here and earlier work, the authors propose a model in which optic lobe glia and neurons constitute a niche for quiescent NPs and that these niche cells respond to injury by using Duox to produce ROS at their cell surfaces. It is further proposed that these ROS activate JNK signalling in both glia and NPs, prompting their re-entry into the cell cycle.

Shortcomings of this work, as well as potential future directions, include reservations regarding the assertion that the authors, ‘ … found broad and perduring JNK activity even 5 days after injury, both in neurons (Elav^+^ cells) and glia (Repo^+^ cells) with levels returning to baseline around 10 days after injury (Figs. 4N–Q and EV4G–H)’. In the published figure (EV4G–H), the upregulation of the JNK reporter is clear, and there is greater upregulation in glia (Fig. 4R), although it is difficult to distinguish between Elav^+^ and Repo^+^ cells or to see whether some of the NPs also upregulate JNK. Clear identification of the cell types that activate JNK will be essential to understanding the molecular mechanisms that activate proliferation. Related to this, glial knockdown of *duox* appears to have a weaker effect on post-injury proliferation than global knockdown, although neither results in a statistically significant increase in injury-induced proliferation (Fig. 2S–T). This raises the possibility that neuronal and/or even NP production of ROS plays a role in activating proliferation, which could be tested with additional cell type-specific knockdowns of *duox* in neurons and NPs. It also is unclear whether ROS or JNK signalling contribute to additional injury responses in the adult brain, including the reprogramming of glia into NPs, the direct reprogramming of glia to neurons, or the recruitment of circulating progenitors. The authors did look for infiltration by haemocytes following injury (Fig. 2L–O) and concluded there is no significant effect. However, given the role of haemocytes in brain regeneration in other arthropods [4], this may be worthy of additional examination.

We suggest a set of experiments that would build upon the work of Alves et al. [1] to further investigate the source and mechanism of proliferation in the adult Drosophila optic lobe. The first of these is designed to ascertain the identities of the proliferating cells following injury in the presence and absence of ROS production. This could be accomplished via immunohistochemistry for a mitotic marker such as phosphohistone H3 (pH3) or for newly synthesized DNA using 5-Ethynyl-2′-deoxyuridine (EdU) in conjunction with labelling for neuronal, glial, and NP markers to reveal the identities of the proliferating cells. A second experiment would evaluate the function of JNK signalling in neurons. Both the global and glial-specific inhibition of JNK signalling via overexpression of *puckered* (*puc*; Figure 4S) showed that JNK signalling is necessary to produce the proliferative response of NPs following injury. It would be interesting to see whether JNK signalling in neurons is also important. This experiment could be conducted using methods already described in the paper, substituting an *elav-GAL4* driver for the *Act5C-GAL4* and *repo-GAL4* drivers used previously. If JNK downregulation in neurons has no effect on the proliferative response following injury, this would support a model in which only glia are providing the proliferative signal to NPs. Alternatively, if JNK downregulation in neurons results in a change to the proliferative response, it could indicate that neurons also contribute to the response via cross-talk, alternative signalling, or other means. Recent evidence does suggest that there is some level of cross-talk among tissue types in the brain [4,5].

Another experiment that would build upon those carried out by Alves et al. [1] is to test the effect of *duox* gain of function in an injured brain. Data presented in Figure 2T demonstrate that *duox* gain of function increases baseline proliferation in uninjured brains; however, parallel data for injured brains were not shown. It remains to be seen whether enhancing Duox activity could boost the regenerative response. Quantifying the numbers of proliferating cells along with the distribution of new neurons and new glia after injury, both with and without an increase in ROS production via overexpression of *duox* would address this question. If more new neurons and/or glia are made following injury when *duox* is overexpressed, this would suggest that enhancing ROS levels by modulating Duox activity could have clinical applications in treating neurodegenerative disorders.

A third set of experiments to extend the work of Alves et al. [1] would examine how other signalling pathways are affected by ROS activation. The *duox-RNAi* experiments establish a model in which to investigate how the optic lobe responds to injury in the presence and absence of glial-derived ROS. An RNA sequencing experiment using this model could identify the signalling pathways that are modulated by changes in ROS levels. For example, it has been reported previously that NPs in the optic lobe are activated by *wingless* signalling [6], and it has been reported that the Toll and IMD pathways play an important role in the proliferative response of the adult central Drosophila brain after injury [5]. Clearly, multiple pathways play a role in the brain’s response to penetrating injury, and it is unlikely that the JNK pathway is uniquely targeted for activation by ROS.

This 2026 paper by Alves et al. [1] provides convincing evidence for the striking observation that glial-derived ROS activate JNK signalling and promote proliferation following injury. Here, we propose future directions for this work aimed at further identifying the cell types and mechanisms involved. We think these experiments will be beneficial in further characterizing the role of ROS in cell proliferation in the adult brain.

## Data Availability

There are no data associated with this paper.
